# Examining the Implementation of the Italian Version of the Teen Online Problem-Solving Program Coupled With Remote Psychological Support: Protocol for a Randomized Controlled Trial

**DOI:** 10.2196/64178

**Published:** 2025-02-21

**Authors:** Claudia Corti, Marta Papini, Sandra Strazzer, Renato Borgatti, Romina Romaniello, Geraldina Poggi, Fabio Alexander Storm, Cosimo Urgesi, Ashok Jansari, Shari L Wade, Alessandra Bardoni

**Affiliations:** 1 Scientific Institute, IRCCS E. Medea Bosisio Parini Italy; 2 Università di Udine Udine Italy; 3 Goldsmiths, University of London London United Kingdom; 4 University of Cincinnati College of Medicine Cincinnati, OH United States

**Keywords:** telerehabilitation, acquired brain injury, executive functioning, pediatric, problem-solving, computer

## Abstract

**Background:**

Pediatric acquired brain injury (ABI) is frequently associated with cognitive and socioemotional alterations. Therefore, targeted rehabilitation to improve everyday functioning, particularly executive functioning (EF), is needed to limit the possible deterioration of cognitive abilities and behavior over time and the associated social and psychological costs.

**Objective:**

In this paper, we present the protocol for a phase-2 randomized controlled trial (RCT) aimed at examining the feasibility and efficacy of a web-based intervention (ie, the Italian version of the Teen Online Problem-Solving [I-TOPS] intervention) to improve problem-solving abilities versus an active-control, web-based intervention (ie, wellness intervention) providing health and wellness content.

**Methods:**

A double-blinded, phase-2 RCT will be conducted to guarantee controls on data quality and findings. In total, 42 adolescents will be recruited from a rehabilitation institute and individually randomly assigned in a 1:1 ratio to receive the I-TOPS intervention or the web-based wellness intervention. Both interventions will include 10 core sessions and will be delivered remotely using a web-based platform. Participants allocated to both interventions and their caregivers will independently complete the learning modules in an everyday setting using their computer. The I-TOPS intervention’s core sessions will target the EF domain (eg, planning, emotion regulation, and social skills), while all the contents of the wellness intervention will be aimed at providing psychoeducation on ABI sequelae and supporting health and wellness. Participants assigned to the I-TOPS intervention will also receive bimonthly direct training in problem-solving coupled with remote support from a psychologist. Feasibility data and efficacy outcomes on both adolescents’ and parents’ functioning will be assessed. Cognitive abilities in the EF domain and behavioral and psychological functioning (ie, internalizing and externalizing symptoms) of the adolescents will be evaluated via performance-based measures, administered remotely using the Google Meet platform, and paper-and-pencil questionnaires; parents’ well-being will be assessed through paper-and-pencil questionnaires. Efficacy will be evaluated immediately after training and at 6-month follow-up.

**Results:**

This study started on February 26, 2021, and ended on February 28, 2023. A total of 42 adolescents were enrolled and randomly assigned to the 2 study groups, 34 (81%) completed the intervention and posttreatment evaluation (I-TOPS: n=19 and wellness intervention: n=15) and 31 performed follow-up evaluation (I-TOPS: n=18 and wellness intervention: n=13). Data analysis on feasibility and efficacy will be performed after protocol publication, and the results will be published in the form of a paper in a relevant journal in 2025.

**Conclusions:**

This double-blinded, phase-2 RCT could extend knowledge on the best rehabilitation practices to adopt with the survivors of pediatric ABI by providing evidence-based data currently lacking for the Italian context. If this study yields positive results, a larger, multicenter, phase-3 RCT could be planned and delivered to examine program cost-effectiveness in a larger sample.

**Trial Registration:**

ClinicalTrials.gov NCT05169788; https://clinicaltrials.gov/study/NCT05169788

**International Registered Report Identifier (IRRID):**

DERR1-10.2196/64178

## Introduction

### Background

Acquired brain injury (ABI), a condition of either traumatic (eg, traumatic brain injury [TBI]) or nontraumatic origin (eg, stroke, anoxia or hypoxia, and infections or inflammation to the brain), represents one of the leading causes of lifelong disability among children and adolescents [1-4]. Together with physical disabilities, the most common consequences of a pediatric ABI include cognitive impairment, reduced academic and vocational attainment, issues with emotional and behavioral regulation, and social problems, all negatively affecting the quality of life of patients [5-15]. Research has indicated that patients with pediatric ABI may be at high risk for substance misuse, mental health problems, criminal behavior, and unemployment in adulthood [5,9,12,15]. Pediatric ABI also substantially causes stress and burden for caregivers and families because children with these conditions experience neurocognitive, behavioral, and adaptive challenges that require relatives to change their lifestyle and develop additional skills to help their children, which often generates significant physical and emotional burden [16,17]. Therefore, pediatric ABI exerts adverse long-term effects on the individual, their family, and the society [6,9,12,15-17].

Specifically, executive functioning (EF) difficulties represent one of the core deficits of ABI, affecting not only cognitive abilities but also behavioral and social functioning [6,9,13,18-24]. EF deficits have been linked with socioemotional adaptation and psychological well-being, with several patients with these deficits exhibiting externalizing behaviors and temper outbursts [6,9,13,18-24]. Thus, there is a clear need for programs designed to rehabilitate EF during the chronic phase of an ABI. However, studies indicate that many children with ABI fail to receive the recommended treatments for optimal recovery after discharge from the hospitals or rehabilitation centers [25-29]. Research has also reported that numerous children with mental health issues do not obtain adequate support [30]. Barriers to receiving treatment have different origins, ranging from intervention accessibility (eg, geographical barriers and time and economic demands to health care facilities and families), poorly designed and grossly underresourced health services, to patients’ and families’ readiness to work on issues [26-31]. Therefore, numerous rehabilitation interventions for this population deliverable at a distance (ie, telerehabilitation) have been developed and offered over the years [31-38]. Telerehabilitation has diminished the financial and time burden on families typically associated with traditional rehabilitation, thereby allowing patients to receive higher therapy doses [31,39-41]. In the field of psychology, telerehabilitation has also been found to reduce the stigma of therapy, to have similar clinical outcomes to traditional face-to-face interventions, and to improve treatment utility and satisfaction [31,39-41].

In the United States, in the mid-2000s, Wade et al [42] developed a technology-assisted intervention for pediatric patients with TBI and, subsequently, with ABI, with the aim to address EF in everyday settings. The program targets problem-solving within the family context, combining the problem-solving framework by D’Zurilla and Nezu [43] and D’Zurilla et al [44] with the collaborative family problem-solving model by Robin and Foster [45]. The Teen Online Problem-Solving (TOPS) program was originally designed with the specific purpose of providing a contextualized treatment that addressed EF, self-regulation, and communication challenges in everyday settings. The web-based format of the intervention had the objective of increasing patients’ participation in outpatient rehabilitation and improving retention rates. The TOPS-contextualized approach is in contrast with training programs that involve only drill-based exercises, which often suffer from a lack of generalization of the effects [37].

The TOPS program includes self-guided, web-based sessions that the child and their family complete at home and bimonthly videoconferences with a psychologist with expertise in cognitive behavioral therapy [42]. The sessions provide training in the steps of problem-solving, teaching adolescents and families to apply them to everyday challenges with organization and planning, social interactions, and emotion control. Metacognitive strategies to promote self-monitoring and self-regulation are also taught. Both the child and the family are required to complete the web-based learning modules, including reading or listening to the didactic information and performing exercises to reinforce understanding of what they read.

In randomized controlled trials (RCTs) of the TOPS program conducted in the United States, families have evaluated the web-based modules of the intervention as helpful, the length and structure as feasible, and the overall program as easy to follow and useful [46,47]. In addition, the web-based program was found to be convenient and beneficial as face-to-face therapy [48]. Regarding efficacy, previous studies have demonstrated the beneficial effects of the TOPS program on children (eg, EF and psychological well-being and behavior at home, school, and other settings); parents (eg, levels of depression and psychological well-being); and even parent-child conflicts [37,42,46,48-52]. In view of this success, recent guidelines acknowledged TOPS as an evidence-based treatment and a standard of care for executive dysfunction and behavioral issues following TBI in the United States [36].

In Italy, there is a great need to develop rehabilitation interventions accessible to large cohorts of patients with ABI, considering the substantial distance of many families from rehabilitation centers and, in some cases, the presence of geographical barriers (eg, living in remote rural areas, mountains, and islands), which make it difficult for many patients to receive treatments in the chronic phase [31,53]. At present, in Italy, telerehabilitation for cognitive and behavioral functions is still at an embryonic phase. The lack of remote interventions for young children with neurological conditions in this country has been highlighted by a review published in 2020 [53], reporting very limited research on the topic, with only one intervention for children with ABI conducted up to 2019 [54]. Importantly, no intervention focusing on everyday EF was detected. In 2020 and 2023, two papers on an RCT of our research group on the effects of a drill-based remote intervention for children with ABI were published, but also in this case, no focus on EF was given [55,56]; no other intervention delivered in Italy was found up to 2023 [57]. Nevertheless, the area of remote interventions is a matter of great interest for the Italian health care system, as suggested by the publication of 2 relatively recent documents on the following topics: the Italian State-Regions agreement on telemedicine delivery published in 2020 [58] and the guidelines for home assistance published in 2022 by the Italian Ministry of Health [59]. These documents report the national guidelines for the provision of remote services and identify the key elements required for the delivery of treatments at a distance.

To increase rehabilitation opportunities for young patients with ABI and embrace the push toward remote interventions in Italy, we translated and adapted the original American TOPS program to create an Italian version of the TOPS (I-TOPS) intervention [31]. The usability of the I-TOPS intervention and its potential positive effects on adolescents with ABI in Italy should be rigorously tested by conducting an RCT, with the aim to inform the Italian health care system on standardized methods to promote recovery and well-being among this population. A clear evaluation of the clinical impact and cost-effectiveness of the program should be provided to support recommendations for effective behavioral and neuropsychological interventions**.**

To this end, a single-center, double-blinded, phase-2 RCT will be conducted, comparing an experimental group receiving the I-TOPS (ie, I-TOPS intervention group) intervention with an active control group receiving a modified version of the program (ie, wellness intervention group), having the same structure but omitting problem-solving–related content. The sample size of the study was established through power analysis by considering data from a previous meta-analytic study on the effects of the original American TOPS program [37]. This paper describes the trial protocol; the steps required to conduct the study, including study procedures (ie, setting, participants’ characteristics, sample size calculation, randomization and blinding, intervention and active control characteristics, and outcomes selected); and a discussion of the clinical implications of the study.

### Objectives

In summary, the study objectives are to (1) examine I-TOPS intervention feasibility in a sample of adolescents with ABI aged 11 to 19 years—we will examine different feasibility outcomes taken from previous studies on remote cognitive rehabilitation interventions for pediatric patients with ABI, considering both the feasibility of the training (eg, accessibility, training adherence, technical smoothness, and training satisfaction) and the feasibility of study and procedures (eg, participation willingness, participation rates, assessment procedures, assessment timescale, and loss to follow-up) [54,60]—and (2) examine I-TOPS intervention efficacy—before training, after training, and at the 6-month follow-up, we will administer questionnaires and performance-based measures to participants and parents in the 2 study groups to assess neurocognitive and psychological or behavioral functioning of children as well as the psychological well-being of parents.

## Methods

### Trial Design

We will conduct a single-center, 2-arm, parallel-group RCT. The trial will apply a pre-post design, with a baseline preintervention assessment (T0), a postintervention assessment immediately after the 6-month intervention period (T1), and a long-term follow-up assessment conducted 6 months after the end of the intervention (T2). Participants will be randomly assigned to groups in a 1:1 ratio to receive the I-TOPS or the wellness intervention. The wellness intervention presents the same structure as the I-TOPS intervention but omits contents related to problem-solving. No change to the I-TOPS or wellness intervention content will be made during the trial; therefore, both interventions will be delivered in their initial Italian version. Eventual failures or system downtimes will be recorded and considered for the evaluation of the feasibility of the training, but no changes in functionality will be made. The trial was registered on ClinicalTrials.gov (NCT05169788) on December 23, 2021.

### Study Setting

The I-TOPS and wellness interventions will be delivered remotely using a web-based platform including web-based learning modules. Participants will complete the intervention in an everyday setting, generally at home using a computer, as the program is not compatible with tablets and mobile phones. Every 2 weeks, a psychologist located in the rehabilitation center (ie, Scientific Institute IRCCS E. Medea in Bosisio Parini, Lecco, Italy) will contact patients and families using the Google Meet videoconference platform to provide remote monitoring, content discussion, and support on the problem-solving process for the I-TOPS intervention and remote monitoring and content discussion only for the wellness intervention.

Demographic, clinical, and outcome data will be collected and stored at the Scientific Institute IRCCS E. Medea.

### Eligibility Criteria

The inclusion criteria for the study, in accordance with those of the studies on the original American TOPS program [42,46,49-52], are as follows: a diagnosis of nonprogressive ABI (eg, TBI, stroke, brain inflammation or infection, and anoxia or hypoxia) in the chronic phase (ie, at least 1 year after the event); ages between 11 and 19 years at the time of the recruitment; full-scale IQ ≥70; proper comprehension and speaking abilities in the Italian language; having a PC and access to the internet in the everyday setting, and adolescent and family familiarity with basic computer and internet literacy to manage emails, access to the internet and websites, and video calls; and at least one parent or guardian living with the adolescent available to participate in the intervention.

Exclusion criteria, in accordance with those of the studies on the original American TOPS program [37,42,46,48-52], are as follows: presence of preinjury or comorbid conditions, such as sensory impairments and global developmental delay; a history of abuse; a history of psychiatric hospitalization; and receiving concomitant psychological intervention.

### Participant Recruitment

The staff members responsible for the study (AB and CC), in conjunction with the referring physicians, will identify potentially eligible participants by reviewing medical records related to pediatric ABIs at the Scientific Institute IRCCS E. Medea. Families of all potentially eligible patients or the patients themselves, if of age, will be met face-to-face in the clinic or contacted by phone by a psychologist of the research team and provided with details about the aims and methods of the study. Parents and adolescents will be given the opportunity to ask questions about the study and to confirm whether they are interested in participating. No information on group allocation, and thus, on differences between the I-TOPS and the wellness intervention will be provided to limit user expectations and biased results; only general information on the structure of the program and its duration and aims will be discussed. If families are interested in participating, eligibility criteria will be checked with the parent to confirm suitability for the research and ascertain whether both parents or which parent will participate with the adolescent during the intervention. A summary of the informed consent will also be provided. To achieve adequate participant enrollment, research assistants will contact all adolescents meeting the inclusion criteria with up-to-date contact information until the target sample size is reached.

### Randomization and Blinding

After completion of the informed consent and baseline measures, participants will be randomly assigned into one of 2 groups: G1, receiving the regular I-TOPS treatment, or G2, receiving the active control training, namely, the wellness treatment. In detail, the randomization will be conducted by a researcher of the Institute, independently from the research staff responsible for recruiting participants. Randomization of patient assignment to the groups will follow a coin flip procedure using the randomization tool of Microsoft Excel: an automated number will be randomly associated with any recruited patient and determine assignment to G1 (0-0.49) or G2 (0.50-1). No stratification will be used. The independent researcher will give the staff members responsible for the study (AB and CC) a sealed envelope containing the participant’s group assignment to keep it concealed from the research staff. This is to avoid selection bias and to prevent the research staff from subverting the allocation sequence. In turn, the research staff will give this envelope to the psychotherapists supervising the interventions, who will contact parents of enrolled adolescents to provide information regarding the next study steps on the basis of the group allocation. Indeed, to allow for clinical supervision, treatment fidelity, and patient interactions, the supervising psychotherapist will be required to receive group allocation information. Other research staff, participants, and testers will remain blinded to group assignment. Given these considerations, this study will constitute a double-blinded RCT. Emergency unblinding will be used only in front of reasonable request by the patient or the referring physician due to clinical reasons.

### Interventions

This study will have 2 treatment arms, that is, the I-TOPS (G1) and wellness (G2) interventions, described subsequently in Textbox 1. Participants will not have to pay or will not be paid to access the allocated intervention.

For both interventions, before starting the training, a psychologist, who will follow training administration, will contact the families via telephone and discuss how to access the treatment sessions on the interventions’ website and how to connect to Google Meet calls. No recommendations on timing, frequency, or intensity of use will be provided; the only requirement will be the completion of each session within a 2-week window. Prompts to use the training will be provided via emails automatically sent by the program; however, participants will choose the frequency (ie, weekly, bimonthly, or monthly) of reminders by selecting a specific option included in the program website. In addition, bimonthly meetings with the psychologist will review content and promote adherence. To this aim, the psychologist will ensure flexibility and coordinate the timing of the sessions to allow families to have time for other commitments and holidays or breaks to be built into the schedule.

No possibility to modify allocated interventions for each trial participant will be given, but each adolescent could discontinue the intervention at any study time due to personal, family, medical, or other reasons.

**Training sessions of the Italian version of the Teen Online Problem-Solving (I-TOPS) intervention and wellness intervention.**

**I-TOPS intervention**

- Session 1 (*Inizio: essere positivi*; Getting started, staying positive, and handling stress): after an introduction on the I-TOPS program and its purposes, the session explains how it is important to develop and maintain a positive attitude in everyday life to better solve problems. The psychologist should review the differences between a positive versus negative approach to problems with the family, emphasizing why it is important to have a positive problem-solving orientation. The family should be encouraged to talk about how they feel when they have problems.
- Session 2 (*Problem-solving*; Problem-solving): the session introduces a 5-step strategy (ie, FAREI, which represents the corresponding acronym to the strategy ABCDE of the original version of the Teen Online Problem-Solving [TOPS] intervention) to solve practical and organizational problems; the 5 steps of problem-solving are as follows: A (ie, aim or focus on a goal), B (ie, brainstorm or analyze all the possible solutions), C (ie, choose a plan), D (ie, do or implement the plan), and E (ie, evaluate what works and what does not work). The psychologist helps the adolescent to apply the strategy by using examples.
- Session 3 (*Organizzarsi*; Getting organized): the session expounds the cognitive changes that occur following an acquired brain injury (ABI), for example, difficulties in memory and attention; the psychologist asks the teenager and the parent about problems with thinking or learning that they feel the teenager is having because of the ABI and about the strategies that they use to compensate for these difficulties. The psychologist helps the family to apply the F.A.R.E.I. strategy learned in session 2 to solve the everyday problems.
- Session 4 (*Lavorare con la scuola*; Working with the school): the session aims to provide parents with information regarding their rights as well as the rights of their adolescent in the education system, with materials and ideas to become advocates for their adolescent, and to create an optimal learning environment while respecting the school’s ideas. In addition, parents are taught strategies to better communicate and work with teachers and are required to solve problems on this topic during the web-based session with the psychologist.
- Session 5 (*Mantenere il controllo*; Staying in control): the session describes the possible behavioral and emotional problems following ABI. The psychologist discusses with the adolescent and their caregivers their own problems to allow them to find a solution. During this session, the adolescent learns the 5-step SMART strategy (which is the same as the one reported in the original TOPS program) useful to empower them to manage their own behavior during social interactions. The 5 steps to stay in control are as follows: S (ie, stop and think), M (ie, monitor your behavior), A (ie, appraise and look at how others are reacting), R (ie, reflect), and T (ie, try a new or different behavior). The psychologist helps the adolescent to apply the strategy in the everyday setting.
- Session 6 (*Gestire la rabbia*; Controlling your anger): the session offers a 6-step strategy to manage anger (ie, SPACCA, which represents the corresponding acronym to the strategy STARRS of the original version of the TOPS intervention) and suggests how to become a good communicator by using *iMessages*. The 6 steps to control anger are as follows: S (ie, stop), T (ie, think about what is happening), A (ie, accept the situation), R (ie, relax), R (ie, reframe or look at the situation differently), and S (ie, solve). The psychologist discusses with the family why a person may have more problems with anger after ABI and helps the adolescent to apply the strategy in the everyday setting.
- Session 7 (*Ascoltare, parlare e leggere i segnali non verbali*; Verbal and nonverbal communication): the session helps the adolescents to focus on verbal and nonverbal communication skills and encourages them to focus on nonverbal signals from communication partners and their own nonverbal communication. The adolescent learns strategies for effective verbal communication. The psychologist supervises the exercises done by the adolescents on the web platform and helps them to identify some situations of everyday life in which the learned abilities could be implemented to improve social communication.
- Session 8 (*Comportamento sociale e di gruppo*; Social behavior and problem-solving): the session focuses on possible everyday difficulties in group settings, romantic relationships, web-based communication, and family context. The psychologist discusses with the adolescent and the family how friendships and social activities could change after an ABI and asks the adolescent to identify social situations that are challenging for them and to apply the strategies learned in sessions 5 and 6 to better deal with others.
- Session 9 (*Prendersi cura di sè*; Taking care of you): the session focuses on the relationship between sleep, nutrition, hydration, physical exercise, and cognitive functioning and wellness following an ABI; it also explains the consequences of feeling stressed and suggests different strategies to manage stress. The psychologist discusses with the family members their own habits and helps the adolescents to use the strategies presented in the session to face difficulties of everyday setting and to maintain an adequate routine to support wellness.
- Session 10 (*Conclusione: mettere insieme i pezzi*; Moving forward and planning for the future): this session recaps the 3 main strategies showed in previous sessions (FAREI, SMART, and SPACCA) and introduces a fourth strategy (FOCUS) devoted at helping adolescents to understand the possible steps to effectively ask for help whenever necessary in view of potential future problems in the everyday setting. This strategy is made of F (ie, flexibility when dealing with something), O (ie, optimism), C (ie, creativity to think about other ways to deal with something), U (ie, use your own resources), and S (ie, support or ask for help). The psychologist helps the adolescent to exercise this fourth strategy to use it in the everyday setting after the end of the training.

**Wellness intervention**

- Session 1 (*La strada verso il recupero*; Recovery and planning for the future): the session introduces the wellness intervention and focuses on the teenager’s global difficulties and worries following an ABI. The psychologist provides psychoeducation on ABI sequelae and encourages the adolescent and their caregivers to describe their personal experience of the injury and related emotions and concerns.
- Session 2 (*Fare fatica è normale*; Acquired brain injuries: possible consequences): the session describes the cognitive changes that can occur following ABI, for example, difficulties in memory, attention, and executive functioning (EF) and includes video testimonials of adolescents with ABI discussing such difficulties. The psychologist encourages the teens and their caregivers to describe their personal experience of the injury and its consequences. The content is aimed at providing psychoeducation on ABI sequelae specifically related to the area of cognition.
- Session 3 (*Essere positivi*; Staying positive and handling stress): the session explains how it is important to hire and maintain a positive attitude in everyday life, but this is not specifically related to the problem-solving process, instead to wellness, reducing perceived stress. The psychologist investigates the family’s use of humor in daily life and encourages them to talk about how they feel and how they react when problems occur.
- Session 4 (*Lavorare con la scuola*; Working with the school): the session aims at providing parents with information regarding their rights as well as the rights of their adolescent in the education system and with materials and ideas to become advocates for their adolescent and to create an optimal learning environment for their adolescent while respecting the school’s ideas.
- Session 5 (*Prendersi cura di sè*; Taking care of you): the session focuses on the relationship between sleep, nutrition, hydration, exercise, and cognitive and physical functioning following ABI. The psychologist investigates with the adolescent and their caregivers their habits in these areas. The aim is to support health and wellness.
- Session 6 (*Gestire lo stress*; Managing your stress): the session explains the consequences of feeling stressed on health and suggests some strategies to manage stress. The psychologist investigates how the adolescents usually cope with stress and helps them to learn the strategies to manage stress and to experiment them in daily life. The aim is to support health and wellness.
- Session 7 (*Il sonno*; Sleep): the session shows the possible sleep changes following ABI and gives advice to improve sleep quality to support health and wellness. The psychologist investigates the adolescent’s sleeping habits and, if necessary, helps them to improve sleep quality. The aim is to support health and wellness.
- Session 8 (*Dopo la scuola superiore*; After high school): the session offers the adolescent an overview on the opportunities provided by the Italian system in terms of education and work activities after high school, with a primarily psychoeducational focus. The psychologist helps the adolescents to think about what they would like to be when they grow up and what paths they could follow.
- Session 9 (*Emozioni di chi si prende cura*; For parents: family coping): the session shows the possible emotional difficulties of caregivers of adolescents with ABI and offers strategies for managing stress within the family context. The psychologist reviews with the caregivers their unique responses to the injury, acknowledging that responses are as unique as they are, with a focus on normalizing their emotional reactions.
- Session 10 (*Parlare con i vostri ragazzi*; Talking with your teenager): the session helps the parents to become good listeners and good communicators to improve their communication with the adolescents and the families to support a calm family environment and overall family wellness. The psychologist engages the parents in role-playing to allow them to improve their communication abilities.

### I-TOPS Intervention (ie, Experimental Intervention Group)

The qualitative data on the process of adaptation of the original TOPS program to the Italian context (ie, I-TOPS) are extensively reported as a case study in a paper published in 2021 [31]. In summary, the adaptation process comprised focus groups with parents of adolescents with TBI to understand the needs of Italian patients and their families; after a literacy translation of the original program into the Italian language, they were asked their opinions on the relevance and clarity of the program content. The main suggestions were related to the scripted videos, with recommendations to emphasizing nonverbal aspects of communication that are integral to the Italian culture. Furthermore, a clearer depiction of the consequences of negative behaviors in the videos was required. The translation of the original TOPS program text from the English language to the Italian language was completed by 3 different psychologists with expertise in the field of brain injury and cognitive behavioral therapy. Translations were subsequently produced by 1 bilingual expert via forward-backward methods, and discrepancies were discussed and resolved. The final text was checked by adolescents with TBI, their parents, physicians, and adolescents of a local school class. Any suggested changes were accepted if they did not alter the meaning of the original sentence. This process required 5 months of work, during which further content revisions were made to improve ease of comprehension and flow in the Italian language.

The regular version of the I-TOPS intervention consists of 10 core sessions focused on problem-solving, EF, behavioral strategies, and social skills and 10 supplemental sessions. Before starting the program, the psychologist will meet with the adolescents and their family to learn about the specific issues occurring in their everyday setting with respect to organization or planning, social interactions, and emotion control that they want to work on during the intervention. Thus, although the I-TOPS program is delivered according to a defined protocol and content, the problem-solving will focus on personalized, everyday issues that the adolescents and their families have identified. The 10 core modules consist of self-guided didactic content regarding the steps of problem-solving and strategies for managing everyday challenges, brief videos modeling these skills, and exercises to support implementation. The adolescents and their families will complete a new module approximately every 15 days. Each web-based session will take 30 to 45 minutes to complete depending on the adolescent’s level of attention. The parent or parents will work with the adolescent to complete each module. The program will not provide any feedback on the activities completed. After the family has completed and reviewed the contents of each session, the trained psychologist will conduct a video meeting of approximately 1 hour with the adolescent and the parent to review the web-based content and practice the problem-solving skills, focusing on a problem identified by the family related to the intervention content from the web-based learning modules (ie, organization or planning, social interactions, and emotion control). Ideally, video meetings should be provided bimonthly, but this period can be slightly adjusted according to family commitments. Completion of all 10 sessions is expected to take each family 5 months. Supplemental sessions will be recommended if their specific content is determined to be potentially beneficial in addressing the ongoing problems of the adolescent or the family identified at the baseline assessment. Therefore, considering supplemental sessions (a maximum of 2 supplemental sessions will be scheduled for each adolescent and family), we anticipate that participants will take approximately 6 months to complete the program.

### Wellness Intervention (ie, Active Control Training)

The active control wellness intervention was developed by translating the original TOPS program contents related to health and wellness into the Italian language, usually included in supplemental sessions, or contents included in original core sessions related to psychoeducation on TBI. In addition, in this case, the translation of the original TOPS program text from the English language to the Italian language was completed by 3 different psychologists having expertise in the field of brain injury and cognitive behavioral therapy. Translations were subsequently produced by 1 bilingual expert via forward-backward methods, and discrepancies were discussed and resolved.

Patients included in the wellness intervention will complete the treatment according to the same schedule as patients of the experimental group but will not receive direct intervention on EF nor support with respect to the problem-solving process. This control treatment focuses on health and wellness but omits contents on problem-solving, EF, behavioral strategies and social skills, and goal setting. The wellness intervention consists of 10 core sessions and 3 supplemental sessions to provide adolescents and their families with a program having the identical structure to the original I-TOPS intervention. The wellness intervention represents an active control training. No feedback will be automatically provided on the activities completed by the program. Bimonthly meetings of a brief duration (ie, about 15 minutes each) with the psychologist will focus on promoting knowledge regarding the consequences of ABI and on healthy lifestyle behaviors to improve wellness and will have the goal to sustain compliance. Completion of all 10 sessions and potentially supplemental ones (a maximum of 2 supplemental sessions will be scheduled for each adolescent and family) is expected to take each family a total of 6 months, similar to the experimental TOPS program. The active control group allows us to control for the placebo effects associated with being involved in a rehabilitation treatment and to isolate the effects of problem-solving training in the I-TOPS intervention.

### Therapist Training

The I-TOPS intervention and the wellness treatment will be delivered by psychologists with expertise in psychotherapy. To ensure fidelity and quality assurance, all psychologists will receive training in recruitment strategies, interviewing techniques, questionnaire administration, and delivery of the I-TOPS and wellness interventions before beginning the program implementation; instructions for delivering each session are detailed in a treatment manual written by the TOPS program developer SLW and her research staff.

### Treatment Fidelity

In relation to the I-TOPS intervention, psychologists will receive weekly supervision from a qualified psychotherapist with a master’s in clinical psychology, a specialization in cognitive behavioral psychotherapy, and qualification in clinical neuropsychology to ensure treatment fidelity and quality assurance.

With respect to the wellness intervention, a supervising psychotherapist will conduct a random review of the sessions conducted by the psychologists every 2 weeks to ensure fidelity. The main topic of discussion will be how to focus on the ABI consequences without directly teaching problem-solving strategies to manage those consequences.

### Data Collection

Following completion of the consent process, sociodemographic information (eg, age, sex, and socioeconomic status); details of past medical history; concurrent medication; the adolescent’s age at the time of injury; and injury severity, measured using the Glasgow Coma Scale [61] will be collected from each patient’s referring physician. Family compositions will be collected from parents via telephone for outpatients or directly in the clinic for hospitalized patients who are close to discharge.

At baseline (T0), after treatment (T1), and at 6-month follow-up (T2), adolescents and parents will be asked to complete study outcome measures, including questionnaires on cognitive and behavioral functioning of adolescents, and the psychological well-being of parents and adolescents will undergo remote cognitive performance–based evaluations. Questionnaires will be sent to adolescents and families in a sealed envelope; after completing them, adolescents and parents will be required to return them to the research staff using an envelope with a postmark included in the sealed envelope within 2 weeks. To promote participant retention, support from a member of the research team could be given for questionnaire fulfillment, if needed. In addition, performance-based measures on adolescents’ cognitive functioning will be remotely administered, thus removing practical barriers to patient retention in the study, such as difficulties in transportation and reaching research centers.

### Feasibility Outcomes

Outcome measures to assess study feasibility will be taken and adapted from previous studies on telerehabilitation interventions for adolescents with ABI, with the aim to use a priori–defined criteria and allow comparisons [54,60]. These measures have been developed for a previous study [60] based on the relevant literature on pilot feasibility studies [62,63]. A total of 9 measures will be used: 4 to assess the feasibility of the I-TOPS or the wellness intervention (ie, accessibility, training adherence, technical smoothness, and training motivation) and 5 to evaluate the study design and procedures (ie, participation willingness, participation rates, assessment procedures, and assessment length and loss to follow-up).

With respect to the feasibility of the I-TOPS or the wellness intervention, the outcomes outlined in Textbox 2 will be considered.

In relation to the feasibility of study design and procedures, the outcomes outlined in Textbox 3 will be considered.

**Outcomes to be considered regarding the feasibility of the Italian version of Teen Online Problem-Solving (I-TOPS) or wellness intervention.**

- Accessibility: number and percentage of participants who ask for further instructions to understand training content and session-related tasks when at home. This criterion refers to understanding how to access the training, how to enter a session, how to perform and save exercises, and how to print specific materials. The criterion for success: 90% of families understand training content and session-related tasks when at home, after the explanation of sessions’ content and activities by the psychologist. For all families, the explanation of the content of sessions will be performed during each scheduled web-based meeting (ie, at the end of each session, the psychologist will briefly explain the content of the following one, also indicating to families the web-based activities they are expected to do for the next meeting), with instructions repeated if a family asks for further clarification. If ≥3 explanations are needed, requiring other remote calls, the criterion will be considered to not have been satisfied. The criterion for success was 100% in the paper presenting the original version of the feasibility criteria [60] and in a previous study of our research group [54], both focusing on remote cognitive interventions having a drill-based format. However, for this study, it was reduced to 90%, given the complexity and multiple requirements of navigating and completing content in the I-TOPS intervention.
- Training adherence: number and percentage of the 10 core sessions completed during the scheduled training period (ie, expected time for completion of the 10 core sessions is 5 months) by each patient, including dropouts. No data on the number of log-ins or average session length will be recorded, as, for both treatments, these data do not provide significant information on better training use and are not considered to be associated with improving target abilities. The criterion for success: average 80% of treatment core sessions is completed after 5 months by each treatment group; the remaining 20% (ie, 2 core sessions) should be completed in a maximum 1 further month (ie, 2 extra weeks for each session). To be adherent, participants needing an additional month to complete the training need to have agreed in advance with the psychologist to postpone sessions due to valid reasons (such as holidays or other family plans). The average completion rate per treatment group in the scheduled time frame, calculated as the sum of the percentage of sessions completed by each participant divided per the total number of participants, is considered.
- Technical smoothness: number and percentage of participants who encounter technical issues with the training material (eg, the platform does not work) that persist for 2 or more weeks, thereby generating a training interruption and possibly influencing total training duration. The criterion for success: all participants will be able to perform the training without technical issues; dropouts are included in the calculation, considering only sessions performed.
- Training satisfaction: training satisfaction will be assessed through a satisfaction questionnaire created ad hoc for the original Teen Online Problem-Solving program and already used in previous studies on its feasibility (Multimedia Appendix 1) [46,47]. The questionnaire assesses perceived helpfulness and usability of the program and improvements as a result of treatment, and for this study, it was translated into the Italian language. Item responses include 1 (ie, strongly disagree), 2 (ie, disagree), 3 (ie, agree), and 4 (ie, strongly agree) on a Likert scale. In particular, 5 items pertain to perceived changes in one’s problem-solving abilities and psychological wellness, 10 items pertain to satisfaction with the overall program (ie, program helpfulness and enjoyment and relevance of contents), and 2 items focus on the website ease of use. The questionnaire generates a total score, with higher scores indicating greater perceived satisfaction. A total score of ≥57 reflects a positive outcome in terms of satisfaction. The criterion for success: 80% of participants have a neutral or positive global score on the questionnaire.

**Outcomes to be considered in relation to the feasibility of the study design and procedures.**

- Willingness to participate: number of participants who accept to partake in the study among those who were contacted for study enrollment. The criterion for success: 75% of eligible participants agree to take part in the study; the criterion is considered on the whole group, as referred to prerandomization data.
- Participation rates: number and percentage of enrolled participants who complete ≥1 session of the interventions, not abandoning the study after baseline measurements and before the start of the training. The criterion for success: 80% of participants who agreed to partake actually participate in the study.
- Assessment procedures: number and percentage of participants for whom outcome data are collected without any problems (eg, missing data and technical issues of assessment tools) on the 3 assessment time points (ie, baseline [T0], after treatment [T1], and 6-month follow-up [T2]). The criterion for success: 90% of the outcome measures for each participant are collected at each time point.
- Assessment timescale: number of participants whose follow-up data are collected within a week after training completion, for posttraining assessment, and within 2 weeks after the 6-month period from training end, for follow-up evaluation. The criterion for success: for all participants, the interval between training end and immediate posttraining evaluation is ≤7 days; for the 6-month follow-up, the assessment is performed in a range of 0 to 14 days from the first day of the 6 months subsequent to training end. This criterion is calculated only for participants performing posttraining and follow-up evaluations, excluding dropouts.
- Loss to follow-up: number of patients who fail to complete all outcome measures at posttraining assessment and 6-month follow-up. The criterion for success: less than 20% of participants enrolled fail to complete all outcome measures on both posttraining assessment and 6-month follow-up. This criterion does not refer to the presence of evaluations with missing data for each participant, for which the criterion “assessment procedures” already provides such information.

### Efficacy Outcomes

A total of 9 outcome measures will be used to test preliminary evidence of training efficacy. Specifically, 7 self-assessed outcome measures (ie, 5 questionnaires for parents and 2 questionnaires for adolescents), 2 performance-based tasks assessing social cognition taken from a standardized neuropsychological battery, and a virtual reality–based assessment of everyday setting EF will be used. Questionnaires will be sent to patients and families in a sealed envelope; after completing them, participants will be required to return them to the research staff using an envelope with a postmark included in the sealed envelope within 2 weeks. The performance-based subtests assessing social cognition and the virtual reality–based assessment will be administered remotely using the Google Meet videoconference platform.

A detailed description of outcome measures is provided in Textbox 4. The sample of this study will include adolescents aged 11 to 19 years, but some measures and questionnaires have been standardized for populations having a lower maximum age range; however, they will be adopted for this study as no other measures with the same characteristics are available in the Italian language. The individual measures affected by this problem are clearly described in Textbox 4.

All outcome measures will be administered at T0 (ie, preintervention assessment), T1 (ie, immediately after the 6-month intervention), and T2 (ie, 6 months after the end of the intervention).

For the questionnaire outcomes, approaches to missing individual items will follow the guidelines for missing item procedures indicated in the manual for each questionnaire. If no guidelines for individual missing items are available, the mean of the completed items will be used to replace missing items if ≤10% are missing.

A summary of outcome measures to test training efficacy is presented in Textbox 5.

**Description of outcome measures.**

- Behavior Rating Inventory of Executive Function Second Edition (BRIEF-2)−parent form [64]: the BRIEF-2–parent form questionnaire is aimed at assessing executive functioning (EF) behaviors of children and adolescents (aged 5-18 years) in everyday life by considering their parents’ perspectives. The questionnaire is composed of 63 items referred to 9 different clinical scales (ie, inhibit, self-monitor, shift, emotional control, initiate, working memory, plan or organize, task-monitor, and organization of materials) and 3 validity scales (ie, inconsistency, negativity, and infrequency). BRIEF 2–parent form is administered to parents, who rate the frequency of executive problems of their children on a 3-point Likert scale. Raw scores on the global scale range from 63 to 189. The *t* scores (mean 50, SD 10) are used to evaluate the level of EF behaviors relative to normative samples. Higher scores indicate a worse outcome. As this questionnaire covers the age range of 5 to 18 years, scores of adolescents of 19 years have been standardized by considering norms for adolescents of 14 to 18 years.
- BRIEF 2–self-report form [64]: the BRIEF 2–self-report form questionnaire is aimed at assessing self-reported EF in everyday life of adolescents aged 11 to 18 years. It is composed of 55 items belonging to 7 clinical scales (ie, inhibit, self-monitor, shift, emotional control, task-monitor, working memory, and plan or organize) and 3 validity scales (ie, inconsistency, negativity, and infrequency). Raw scores on the global scale range from 55 to 165. The *t* scores (mean 50, SD 10) are used to interpret the level of EF compared to normative sample. Higher scores represent a worse outcome. As this questionnaire covers the age range 5 to 18 years, scores of adolescents of 19 years have been standardized by considering norms for adolescents of 14 to 18 years.
- Child Behavior Checklist 6 to 18 (CBCL 6-18) [65]: the CBCL 6 to 18 is aimed at assessing psychological adjustment and behavioral functioning of children and adolescents aged 6 to 18 years, as rated by parents. The questionnaire includes 113 items. The scores of this instrument considered for this study are total problems, internalizing problems, and externalizing problems. Raw scores of the Total Problems Scale range from 0 to 226 and are converted to *t* scores (mean 50, SD 10) to interpret the level of behavioral functioning compared to normative sample. Higher scores indicate a worse outcome. As this questionnaire covers the age range 6 to 18 years, scores of adolescents aged 19 years have been standardized by considering norms for adolescents aged 12 to 18 years.
- Youth Self-Report 11 to 18 (YSR 11-18) [65]: YSR 11 to 18 is aimed at assessing self-reported psychological adjustment and behavioral functioning of adolescents aged 11 to 18 years. The questionnaire includes 112 items. The scores of this instrument considered for this study are total problems, internalizing problems, and externalizing problems. Raw scores of the Total Problems Scale range from 0 to 224 and are converted to *t* scores (mean 50, SD 10) to interpret the level of behavioral functioning compared to normative sample. Higher scores represent a worse outcome. As this questionnaire covers the age range 11 to 18 years, scores of adolescents aged 19 years have been standardized by considering norms for adolescents aged 11 to 18 years.
- Beck Anxiety Inventory (BAI) [66]: the BAI is a 21-item questionnaire aimed at assessing state and trait anxiety in adults. In this study, the questionnaire is administered to parents to evaluate their psychological functioning. The total score is calculated as the sum of the 21 items (4-point Likert scale, ranging 0-3), with a minimum score of 0 and a maximum score of 108. Higher scores indicate a worse outcome. Specifically, a score of 0 to 21 indicates low levels of anxiety, a score of 22 to 35 indicates moderate levels of anxiety, and a score of 36 to 108 indicates potentially concerning levels of anxiety.
- Symptom Checklist 90 (SCL-90) [67]: the SCL-90 is a self-report questionnaire for adults aimed at measuring psychiatric symptom intensity on 9 different subscales (ie, somatization, obsessive-compulsive, interpersonal sensitivity, depression, anxiety, anger-hostility, phobic anxiety, paranoid ideation, and psychoticism). The 90 items included in the questionnaire are scored on a 5-point Likert scale (ranging 0-4), indicating the rate of occurrence of the symptoms during the last 7 days. In this study, the questionnaire is administered to parents to assess their psychological well-being. The Global Severity Index (GSI) is considered, for which the score is reported as a ratio of the sum of all items to the number of items scored. Higher scores indicate higher psychiatric symptom intensity; consistent with recommendations [65], an average score corresponding to ≥1 on the GSI enters the clinical range.
- Parenting Stress Index (PSI)–Short Form [68]: PSI–Short Form is a 36-item questionnaire aimed at assessing levels of stress associated with parenting in relation to different areas: parenting competence, restrictions on life introduced by parenting, parental conflict, depression, and social support. The 36 items of the questionnaire are scored on a 5-point Likert scale. The global score (PSI-total) is considered for this study, for which clinical cutoff is established at 90. Higher scores indicate higher distress.
- Jansari assessment of Executive Functions for Adolescents (JEF-A) [69]: JEF-A is a computerized assessment based on nonimmersive virtual reality aimed at evaluating everyday EF in adolescents aged 10 to 18 years. It is a performance-based assessment administered to adolescents. Participants are asked to plan, set up, and run a birthday party through the completion of 16 tasks resembling real-world activities. JEF-A tasks evaluate 8 constructs related to EF: planning, prioritization, selective thinking, creative thinking, adaptive thinking, action-based prospective memory, event-based prospective memory, and time-based prospective memory. All tasks are scored on a 3-point scale: 0 for failure, 1 for a partial or nonoptimal completion, and 2 for satisfactory completion. The scores for the 2 tasks for any particular construct are summed (ie, maximum of 4 possible), and this score is converted to a percentage of achievement for this construct. In addition to the 8 individual module scores, an average total percentage is computed for the whole assessment. The final raw score ranges from 0 to 32. Higher scores and percentages indicate a better EF. Although this instrument has been tested on adolescents with acquired brain injury (ABI) who are aged 10 to 18 years, in this study, we adopted it for participants aged 19 years as well to ensure comparability and a task in line with tasks related to such an age instead of administering to those adolescents the task for adults involving a multiple errand task based around a business office [70].
- A Developmental Neuropsychological Assessment-2 (NEPSY-2; Theory of Mind and Affect Recognition subscales) [71]: the Theory of Mind and Affect Recognition subscales of NEPSY-2 are performance-based subtests administered to adolescents and aimed at evaluating their social perception and emotion recognition skills. Theory of Mind subscale–part A measures understanding of mental functions and other people’s perspectives, and its raw scores range from 0 to 17; Theory of Mind subscale–part B examines the ability to match basic emotions (eg, happy, sad, angry, afraid, and disgusted) to specific situations, and its raw scores range from 0 to 8. The Affect Recognition subscale tests the ability to recognize emotions in facial expressions, and its raw scores range from 0 to 35. Raw scores are converted into scaled scores (mean 10, SD 3) based on age norms ranging from 1 to 19. Higher scores indicate better outcomes. As this battery covers the age range 3 to 16 years, scores of adolescents aged 17 to 19 years have been standardized by considering norms for adolescents aged 11 to 16 years.

**Outcome measures to test training efficacy administered to adolescents and parents.**

**Cognitive outcomes**

- Jansari assessment of Executive Functions for Adolescents (JEF-A; performed by the adolescent) [69]
- A Developmental Neuropsychological Assessment-2 (NEPSY-2; performed by the adolescent) [71]
- Behavior Rating Inventory of Executive function 2 (BRIEF 2-self-report form; related to self-functioning. filled by the adolescent) [64]
- Behavior Rating Inventory of Executive function 2 (BRIEF 2-parent form; related to the adolescent, filled by the parent) [64]

**Behavioral or psychological outcomes**

- Youth Self-Report 11 to 18 (YSR 11-18; related to self-functioning, filled by the adolescent) [65]
- Beck Anxiety Inventory (BAI; related to self-functioning; filled by the parent) [66]
- Child Behavior Checklists 6 to 18 (CBCL 6-18; related to the adolescent, filled by the parent) [65]
- Parenting Stress Index (PSI; related to self-functioning, filled by the parent) [68]
- Symptom Checklist-90 (SCL-90; related to self-functioning, filled by the parent) [67]

### Sample Size and Power Calculation

A previous meta-analysis on the effects of the original American TOPS program on the various outcomes found a small-to-medium average effect size (Hedges *g*=0.37) [37]; however, high variability was found among the effect sizes of the different studies analyzed. This led us, to be conservative, to estimate a small effect size of f=0.2 for the evaluation of the power calculation in the present study. Power analysis was conducted using G*Power3 Software (Heinrich-Heine-Universität Düsseldorf) [72,73]. Assuming a correlation of 0.50 between repeated measures and setting the α level at *P*<.05, a sample size of 21 participants per group will be required to obtain 80% of power with our 2 group- and 3 time-point- design. Therefore, a whole sample of 42 participants will be required to be included in the study. Attrition was not considered in performing power calculation, as adherence rates will be a core outcome of this study, which is pioneering for the Italian context and, therefore, will be aimed at collecting data on feasibility. Therefore, any eventual dropouts will be accounted for in statistical analyses using intention-to-treat analysis procedures.

### Data Analysis Plan

All randomized adolescents will be included in the analyses, irrespective of adherence to treatment in the I-TOPS or wellness intervention. No imputation of a missing baseline or follow-up data will be performed. The *t* tests and chi-square analyses will be performed to compare the I-TOPS (ie, experimental) and wellness (ie, control) intervention groups on continuous and dichotomous variables, respectively. Similar analyses will be performed to examine baseline differences between patients who will complete the study and those who will drop out. Frequencies and means related to feasibility outcomes will be calculated. Analyses will be performed using SPSS (version 29.0.1.0; IBM Corp) [74].

Efficacy outcome measures will be separately entered into linear mixed effect models with “time” (baseline vs immediately after treatment vs 6-month follow-up) and “treatment” (regular I-TOPS vs wellness intervention) and their interaction as fixed factors and “intercepts” and “participants” as random factors. In case of differences in any clinical or demographic variables between groups at baseline, those measures will be included in the analyses as covariates. Mixed model analysis allows flexible modeling of the pattern of change over time and uses all the data for a given participant, even if that participant is not seen at all assessments, allowing us to retain participants with missing assessments. Significant interaction effects (ie, time and group) or group effects will be analyzed by post hoc tests. The significance threshold will be set at <.05. No interim analysis will be planned.

### Safety Reporting

The risks associated with participating in this trial are considered minimal. The exclusion of adolescents with photosensitive epilepsy reduces the possible risks associated with the prolonged use of a technological device. The interventions, both the original I-TOPS and the wellness interventions, could raise awareness of injury-related cognitive or behavioral problems, which could contribute to conflict between family members. Family communication and problem-solving in the I-TOPS group might increase family burden. Nevertheless, the purpose of the original I-TOPS program is ultimately to help families cope with these difficulties by teaching problem-solving and communication strategies. In addition, the psychologist supporting the web-based interventions (ie, both original I-TOPS and wellness intervention) is trained to handle any emerging problems. Should any issues arise, the psychologist will have the opportunity to discuss the situation during weekly group supervision meetings with the supervising psychotherapist who is qualified in psychotherapy (a minimum of 2 psychologists is scheduled to deliver the treatments). Thus, there is no requirement to report nonserious adverse events (SAEs) in this study; nevertheless, a monitoring of SAEs will be performed. SAEs may be reported by referring physicians of each patient or researchers, the I-TOPS intervention coaches, participants themselves, or any other informant. Adverse event data will be monitored by the Ethics Committee of the Scientific Institute, IRCCS E. Medea to ensure safety. All suspected SAEs will be reported within 24 hours of discovery to the chief investigator, who will be asked to inform the ethics committee contact person at our Institute. All SAEs will be followed up until resolution.

### Ethical Considerations

This study has been approved by the Ethics Committee of Scientific Institute, IRCCS E. Medea (08/21-CE, January 21, 2021). The trial will be conducted in accordance with the protocol and the principles of the 1964 Declaration of Helsinki. Any eventual amendments to the protocol will be submitted to the Ethics Committee of Scientific Institute, IRCCS E. Medea for approval. The principal investigator should make available, on request, relevant trial-related documents for monitoring and audit by the sponsor (Scientific Institute, IRCCS E. Medea) or the relevant research ethics committee.

The informed consent will be obtained face-to-face by staff members responsible for the study (AB and CC) when the adolescents and parents come to the clinic or remotely by using a sealed envelope for those families not able to reach the Institute. The consent process depends on the age of the participants; for participants aged 11 to 17 years, the parents or guardians will provide consent, while participants aged 18 or 19 years will provide their own signed consent. Families will be required to sign the “participant information sheet and informed consent for research participation.” Participants will be given the opportunity to express their concerns and declare barriers to participating in the study at the time of enrollment. Participants will have the ability to opt out at any time during the study.

Parents of participants below the age of consent or participants of age will be required to sign a “privacy policy statement and consent.” An Institute Data Protection Officer (DPO) will be available, representing a contact point between the Italian Data Protection Authority and the concerned parties. DPO email address is reported on the “privacy policy statement and consent” signed by the parents or the participants. In addition, using the email address of the DPO reported on the “privacy policy statement and consent,” participants will have the possibility to provide feedback and express any concerns about the project.

Participants’ personal data will be pseudonymized. A unique trial number will be provided to each participant consenting to participate in the study. Each participant will be identified in all study-related documentation by the trial number and initials. A record of names and addresses linked to participants’ trial numbers will be maintained by the research team and stored securely. All data will be entered into a password-protected database and encrypted using a stored procedure. Data will be stored for 50 years, except in the case of extension by law, in accordance with information reported in the document on data management for scientific research.

To prevent problems with mislaid usernames and passwords, participants will access the web pages of the allocated training program through links emailed to them by the research team members. Once the program has been completed by the participant, it will be locked to prevent further data entry or change.

The trial database will be designed and maintained by 2 members of the research team responsible for data management (AB and CC); access to the database will be given to eventual research assistants involved in the research project by signaling their name to the specific privacy office of the Institute. Database access by researchers will be password protected.

All data will be collected and managed in accordance with Regulation (European Union) 2016/679 of the European Parliament and of the Council of April 27, 2016, on the protection of natural persons with regard to the processing of personal data and on the free movement of such data and repealing Directive 95/46/EC.

No compensation related to study participation will be provided.

## Results

After the approval of the ethics committee, the study started on February 26, 2021, and ended on February 28, 2023. Recruitment started on December 27, 2021, and ended on February 25, 2022. The last patient evaluation was performed on February 22, 2023. A secular event, namely the COVID-19 emergency, fell into the first part of the study period, which could have increased the interest of adolescents and families in participating in a remotely delivered treatment. However, due to the need of the target population to receive neurocognitive treatment in the chronic phase, we consider this fact marginal and not influencing the adherence rate. At study protocol submission in *JMIR Research Protocols*, the study has been concluded; the expected 42 participants were randomly assigned into the 2 treatment groups, and 34 (81%) of them concluded the assigned intervention and underwent posttraining and 6-month follow-up evaluations. The total attrition rate was 19% (8/42).

**Figure 1:**
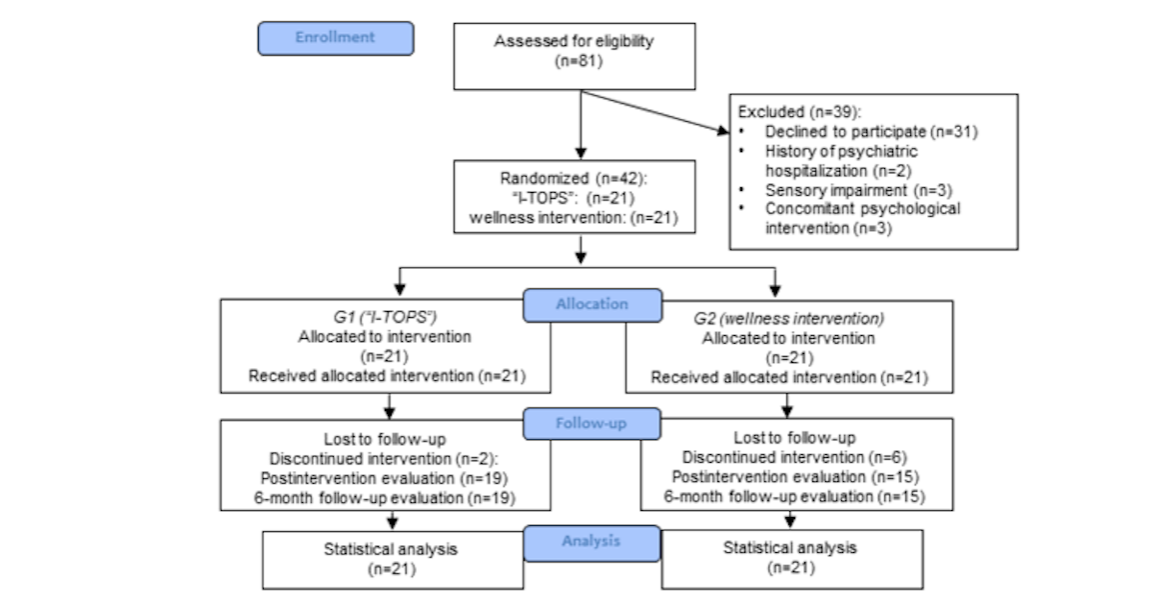
CONSORT (Consolidated Standards of Reporting Trials) flow diagram. I-TOPS: Italian version of teen online problem-solving.

Figure 1 depicts the CONSORT (Consolidated Standards of Reporting Trials) flow diagram.

After the publication of the protocol, data analysis to test feasibility and efficacy will be performed, and final results are expected to be published in the form of a paper in a relevant scientific journal in 2025. In this paper, demographics associated with digital divide issues such as age, education, gender, family socioeconomic status, and computer or internet literacy will be reported.

## Discussion

### Principal Findings

This RCT will evaluate the feasibility and efficacy of a web-based training aimed at improving EF and problem-solving abilities (ie, I-TOPS intervention) versus an active control web-based intervention providing health and wellness content (ie, wellness intervention) in adolescents with ABI. Substantial evidence on the feasibility and efficacy of the original TOPS program developed and delivered in the United States has been collected, while this RCT will be the first study providing data for the Italian context using the adapted Italian version of the intervention (ie, I-TOPS).

EF rehabilitation is highly recommended for pediatric patients with ABI to limit the progressive deterioration of behavior over time and to reduce associated social and psychological costs [31,75]. Telerehabilitation represents a new form of service that mitigates issues associated with face-to-face rehabilitation, such as limitations in access or elevated costs for families and hospitals, creating more care opportunities for patients. Until now, in Italy, no specific remote intervention for EF for this population is available [57]. Therefore, the TOPS program, originally developed in the United States to help teenagers with TBI and, subsequently, other types of ABI to improve problem-solving in the everyday setting, has been translated and adapted to the Italian cultural context with the name “I-TOPS.” The I-TOPS intervention urgently needs to be administered in a rigorous RCT in Italy, allowing a test of its feasibility, clinical efficacy, and cost-effectiveness. Our research team has already conducted pilot trials on telerehabilitation treatments for children with ABI in recent years and has also concluded and published a phase-2 RCT on the effects of a drill-based cognitive training [55,56]. However, data on the efficacy of the program were promising only for visual-spatial working memory and not for other cognitive domains, including EF or behavioral and psychological measures [55,56]. Therefore, at a clinical level, the need remains to provide patients with a rehabilitation program to improve EF in everyday settings. This research is the first Italian study that will focus on this topic.

We will conduct a phase-2 RCT to guarantee adequate controls on data quality and findings, consistent with recommendations proposed for studies on rehabilitation interventions [75]. This RCT protocol is reported in accordance with the CONSORT-EHEALTH (Consolidated Standards of Reporting Trials of Electronic and Mobile Health Applications and Online Telehealth) checklist (version 1.6.1) [76] (Multimedia Appendix 2), which allows for improving the quality of reports of web-based intervention evaluations. Data from this RCT will help inform future directions for investments in the field of telerehabilitation in Italy.

### Strengths and Limitations

The planned study possesses a number of strengths, including the use of an active control group (ie, wellness intervention) instead of a passive one, which allows controlling for placebo effects. Furthermore, although the I-TOPS program involves self-directed, web-based learning modules, the provision of active problem-solving therapy by a trained cognitive behavioral psychologist constitutes a key aspect for the success of this RCT. In support of this, a recent study highlighted the fundamental role of patient-therapist engagement in obtaining good results during the rehabilitation therapy [77]. Finally, the use of the computer, rather than a smartphone, for treatment delivery should allow adolescents to maintain an adequate level of commitment and concentration while performing the I-TOPS intervention, requiring them and their families to carry out the program in a quiet place at home and not in random places (eg, on the beach and means of transportation). However, at the same time, this requirement may restrict treatment access and preclude participation by families who do not have the financial means to buy a computer and the necessary equipment for the videoconferences. Furthermore, families who go on vacation during the 6-month intervention period will have to plan to bring a computer to continue the program or postpone appointments if they are unable to access a computer, which may cause problems with respect to the treatment schedule. At the same time, the long study duration could cause study adherence issues. Nevertheless, it will be the responsibility of our research team to indicate whether the postponement of the sessions is agreed in advance (ie, for holidays or other justified or warranted engagements of the family) or it is instead linked to other factors associated with feasibility issues (eg, forgetfulness, lack of desire or motivation, and failure to carry out tasks). An accurate examination of the feasibility associated with both the training and study design and procedures will be reported.

To limit the issues related to potential biases associated with subjective measures [37], this study examines efficacy as well by using performance-based measures. Furthermore, it adopts a virtual reality–based assessment [69] requiring adolescents with ABI to do errands typical of the everyday setting, which could provide important information on their actual functioning in activities similar to those of real life. Inclusion of these measures will provide further evidence regarding the utility of the intervention in improving various neuropsychological consequences, and thus, this information could be useful to provide the National Health Care System with recommendations for survivors of pediatric ABI.

In addition, the examination of training and study procedure feasibility will allow investigating the usability of the training and the adequateness and potential replicability of the study design, which could help addressing potential issues to intervention delivery in the clinical context and study replication by future research teams. This could also favor the evaluation of the generalizability of trial findings to the general patient population of adolescents with brain injury, addressing sources of potential bias and imprecision. In the clinical context outside of an RCT setting, the I-TOPS intervention would be delivered in the same exact way as in this study, in terms of content, prompts to use the training and human involvement, which increases the relevance of findings of this study to inform the clinical practice.

### Conclusions

If this phase-2 RCT yields positive results, a larger, multicenter, phase-3 RCT could be planned and delivered to examine I-TOPS intervention efficacy and cost-effectiveness in a larger sample. Since 2020, Italy has a network for sharing the best practices related to rehabilitation for pediatric neurological children—Rete Pediatrica degli IRCCS [78]—with an important focus on telerehabilitation. This network could enroll in the phase-3 RCT, particularly given that the institute conducting the phase-2 RCT is part of this network.

If the phase-3 RCT demonstrates efficacy, future work will focus on broader implementation within the Italian health system. Beyond providing clinical benefits to adolescents with ABI and their families, the I-TOPS intervention might reduce longer-term health care service use, thus limiting rehabilitation costs. The program might also have broader societal benefits through improvements in educational and vocational outcomes and reductions in criminal behavior frequently associated with EF impairments following ABI [6,9,13,18-24]. Considering local policy making and treatment delivery, the I-TOPS intervention requires limited economic resources to be implemented due to its web-based format and allows for a centralized psychologist, with expertise in the program and pediatric ABI, to provide treatment throughout the country rather than limited to a single region. Therefore, I-TOPS intervention delivery does not necessarily need individuals with this expertise throughout Italy, especially in rural areas or islands, but could allow clinicians of specialized rehabilitation centers to take charge of a wide range of patients, also reaching those living in remote regions. In addition, with the program being accessible at any time that is convenient for the adolescents and their families, it could be less burdensome than traditional clinic appointments, which often require children to miss school lessons or parents to modify their work schedules. Using I-TOPS intervention materials, the National Healthcare System might routinely provide the program to adolescents with ABI and their families throughout the country using a single central therapist to support the needs of multiple families in disparate locations. The findings from this RCT will be disseminated through publications in peer-reviewed and popular science journals and presentations at scientific conferences with the aim to reach relevant research, clinical, health service, and patient communities.

## Appendix

Multimedia Appendix 1 Training satisfaction questionnaire.

Multimedia Appendix 2 CONSORT-eHEALTH checklist (V 1.6.2).

## Abbreviations

ABI: acquired brain injury
CONSORT: Consolidated Standards of Reporting Trials
CONSORT-EHEALTH: Consolidated Standards of Reporting Trials of Electronic and Mobile Health Applications and Online Telehealth
DPO: Data Protection Officer
EF: executive functioning
I-TOPS: Italian version of Teen Online Problem-Solving
RCT: randomized controlled trial
SAE: serious adverse event
TBI: traumatic brain injury
TOPS: Teen Online Problem-Solving
