# Supplementary material for: Examining the Implementation of the Italian Version of the Teen Online Problem-Solving Program Coupled With Remote Psychological Support: Protocol for a Randomized Controlled Trial
Source: JMIR Res Protoc. 2025 Feb 21;14:e64178. doi: 10.2196/64178 (PMC11890147; doi:10.2196/64178)
Supplement: Multimedia Appendix 1 [file resprot_v14i1e64178_app1.pdf]

Questionario di soddisfazione sul training
[Training satisfaction questionnaire]

	Fortemente in disaccordo [*Strongly Disagree*]	Disaccordo [*Disagree*]	Accordo [*Agree*]	Fortemente d'accordo [*Strongly Agree*]
1. Ho raggiunto gli obiettivi che mi ero posto/a a inizio intervento [*I have reached the goals that I had when I began the program*].	1	2	3	4
2. Ho una strategia per gestire i problemi futuri [*I have a plan for handling future problems*].	1	2	3	4
3. So come gestire una crisi se/quando mi capiterà [*I know how to handle a crisis when it comes up*].	1	2	3	4
4. Mi sento meno stressato/a [*I feel less stressed*].	1	2	3	4
5. L'intervento è stato troppo lungo [*The program was too long*].	1	2	3	4
6. L'intervento ha avuto una lunghezza adeguata [*The program had an appropriate length*].	1	2	3	4
7. Le informazioni contenute nel programma non sono state utili a me/alla mia famiglia [*The information included in the program did not apply to me or my family*].	1	2	3	4

8. Rifarei l’intervento<br>[*I would do the program over*].	1	2	3	4
9. Consiglierei a qualcun altro di partecipare all’intervento<br>[*I would recommend the program to others*].	1	2	3	4
10. Ho fatto dei buoni cambiamenti<br>[*I have made positive changes*].	1	2	3	4
11. L’intervento è stato come me lo immaginavo<br>[*The program met my expectations*].	1	2	3	4
12. L’intervento è stato d’aiuto<br>[*The program was helpful*].	1	2	3	4
13. L’intervento è stato utile<br>[*The program was useful*].	1	2	3	4
14. L’intervento mi è piaciuto<br>[*I enjoyed the program*].	1	2	3	4
15. Il programma è stato facile da usare<br>[*The website was easy to use*].	1	2	3	4
16. Il sito web è stato utile<br>[*The website was useful*].	1	2	3	4

17. Mi è piaciuto utilizzare il sito web<br>[*I enjoyed using the website*].	1	2	3	4
18. Il sito web è stato facile da capire e utilizzare<br>[*The website was easy to understand and navigate*].	1	2	3	4
19. I contenuti sono stati interessanti<br>[*The content was relevant*].	1	2	3	4
