# Supplementary material for: Examining the Implementation of the Italian Version of the Teen Online Problem-Solving Program Coupled With Remote Psychological Support: Protocol for a Randomized Controlled Trial
Source: JMIR Res Protoc. 2025 Feb 21;14:e64178. doi: 10.2196/64178 (PMC11890147; doi:10.2196/64178)
Supplement: Multimedia Appendix 2 [file resprot_v14i1e64178_app2.pdf]

CONSORT-EHEALTH Checklist V1.6.2 Report	Manuscript Number	64178
**(based on CONSORT-EHEALTH V1.6), available at [http://tinyurl.com/consort-ehealth-v1-6].**		
**Date completed**		
11/15/2024 11:36:12		
**by**		
Claudia Corti		
TITLE		
**1a-i) Identify the mode of delivery in the title**		
**1a-ii) Non-web-based components or important co-interventions in title**		
title: "Implementation of the Italian Version of the web-based Teen Online Problem-Solving (TOPS) Program coupled with remote psychological support via video meetings for adolescents with acquired brain injury: A Protocol for a Randomized Control Trial"		
**1a-iii) Primary condition or target group in the title**		
title: "Implementation of the Italian Version of the web-based Teen Online Problem-Solving (TOPS) Program coupled with remote psychological support via video meetings for adolescents with acquired brain injury: A Protocol for a Randomized Control Trial"		
ABSTRACT		
**1b-i) Key features/functionalities/components of the intervention and comparator in the METHODS section of the ABSTRACT**		
**1b-ii) Level of human involvement in the METHODS section of the ABSTRACT**		
"Forty-two adolescents will be recruited from a rehabilitation institute and individually randomized in a 1:1 ratio to receive the online I-TOPS intervention or the online wellness intervention. Both interventions will include 10 core sessions and will be delivered remotely using a web-based platform. Participants allocated to both interventions and their caregiver(s) will independently complete the learning modules in an everyday setting using their computer."<br>For further information see manuscript, heading "Methods" of the Abstract section.		
**1b-iii) Open vs. closed, web-based (self-assessment) vs. face-to-face assessments in the METHODS section of the ABSTRACT**		
"Participants assigned to the I-TOPS intervention will also receive bimonthly direct training in problem-solving coupled with remote support from a psychologist."		
**1b-iv) RESULTS section in abstract must contain use data**		
"Forty-two adolescents will be recruited from a rehabilitation institute..."; "Cognitive abilities in the EF domain and behavioral and psychological functioning (internalizing and externalizing symptoms) of the adolescents will be evaluated via performance-based measures, administered remotely using the Google Meet platform, and paper-and-pencil questionnaires; parents well-being will be assessed through paper-and-pencil questionnaires."		
**1b-v) CONCLUSIONS/DISCUSSION in abstract for negative trials**		
"This study started on February, 26, 2021 and ended on February, 28, 2023. A total of 42 adolescents were enrolled and randomized into the two study groups (I-TOPS vs wellness intervention), and 34 adolescents (19 I-TOPS and 15 wellness) completed the intervention and post-treatment and follow-up evaluations. Data analysis on feasibility and efficacy will be performed after protocol publication and results will be published in the form of a paper in a relevant journal in 2025."		
INTRODUCTION		
**2a-i) Problem and the type of system/solution**		
**2a-ii) Scientific background, rationale: What is known about the (type of) system**		
"Specifically, executive functioning (EF) difficulties represent one of the core deficits of ABI, affecting not only cognitive abilities, but also behavioral and social functioning [18-25]. EF deficits have been linked with socio-emotional adaptation and psychological well-being, with a number of patients suffering from these deficits exhibiting externalizing behaviors and temper outbursts [19-25]. Thus, there is a clear need for programs designed to rehabilitate EF during the chronic phase of an ABI."<br>"In Italy there is a great need to develop rehabilitation interventions accessible to large cohorts of patients with ABI, considering the substantial distance of many families from rehabilitation centers and, in some cases, the presence of geographical barriers (such as living in remote rural areas, mountains and islands), which make it difficult for many patients to receive treatments in the chronic phase [32,52]."		
METHODS		
**3a) CONSORT: Description of trial design (such as parallel, factorial) including allocation ratio**		
**3b) CONSORT: Important changes to methods after trial commencement (such as eligibility criteria), with reasons**		
"To this end, a single-center, double-blinded, phase II RCT will be conducted, comparing an experimental group receiving the I-TOPS (I-TOPS group) with an active control group receiving a modified version of the program (wellness intervention group), having the same structure but omitting problem-solving related content."<br>"In sum, study objectives are:<br>-to examine I-TOPS feasibility in a sample of adolescents with ABI aged 11-19 years: we will examine different feasibility outcomes taken from previous studies on remote cognitive rehabilitation interventions for pediatric patients with ABI, considering both the feasibility of the training (accessibility, training adherence, technical smoothness, and training satisfaction) and the feasibility of study and procedures (participation willingness, participation rates, assessment procedures, assessment timescale and loss to follow-up) [53,59].<br>-to examine I-TOPS efficacy: at pre-training, post-training and 6-month follow-up we will administer to participants and parents of the two study groups questionnaires and performance-based measures on neurocognitive and psychological/behavioral functioning of children and psychological well-being of parents."		
**3b-i) Bug fixes, Downtimes, Content Changes**		
**4a) CONSORT: Eligibility criteria for participants**		
"A single-center, two-armed (parallel groups) RCT will be conducted. The trial will apply a pre-post design, with a baseline pre-intervention assessment (T0), a post-intervention assessment immediately after the 6-month intervention period (T1) and a long-term follow-up assessment conducted 6 months after the end of the intervention (T2). Participants will be randomly assigned to groups in a 1:1 ratio to receive the I-TOPS or the wellness intervention."		
**4a-i) Computer / Internet literacy**		
**4a-ii) Open vs. closed, web-based vs. face-to-face assessments:**		
"-having a personal computer and access to the Internet in the everyday setting and adolescent and family familiarity with basic computer and Internet literacy to manage emails, access to the Internet and web-sites and video-calls".		
**4a-iii) Information giving during recruitment**		
"The staff members responsible for the study (A.B. and C.C.), in conjunction with the referring physicians, will identify potentially eligible participants, by reviewing medical records related to pediatric ABIs at Scientific Institute IRCCS E. Medea, Bosisio Parini, Lecco, Italy. Families of all potentially eligible patients or the patients themselves (if of age) will be met face-to-face in the clinic or contacted by phone by a psychologist of the research team and provided with details about the aims and methods of the study.";<br>"In addition, bimonthly meetings with the psychologist will review content and promote adherence. To this aim, the psychologist will ensure flexibility, coordinating the timing of the sessions to allow families to have time for other commitments and holidays/breaks to be built into the schedule."<br>"The performance-based subtests assessing social cognition and the virtual-reality based assessment will be administered remotely using the Google Meet platform."		
**4b) CONSORT: Settings and locations where the data were collected**		
"No change on the I-TOPS or wellness intervention content will be made during the trial, therefore both interventions will be delivered in their initial Italian version."		
**4b-i) Report if outcomes were (self-)assessed through online questionnaires**		
**4b-ii) Report how institutional affiliations are displayed**		

The item is not applicable to the manuscript as no online questionnaires were used.		
**5) CONSORT: Describe the interventions for each group with sufficient details to allow replication, including how and when they were actually administered**		
**5-i) Mention names, credential, affiliations of the developers, sponsors, and owners**		
**5-ii) Describe the history/development process**		
"The authors declare that they have no competing interests. Dr. Shari Wade, an author of this paper, is the developer of the original TOPS program, which was adapted for the Italian context (I-TOPS) by the first author, Dr. Claudia Corti. Related to this aspect, we declare that no conflict of interest exists in data reporting: Dr. Claudia Corti was not responsible for patient enrollment and allocation and was masked with respect to participants' group allocation; Dr. Shari Wade did not have access to any information on patients nor to the database."		
**5-iii) Revisions and updating**		
"In the USA, in the mid2000's, Wade and colleagues developed a technology-assisted intervention for pediatric patients with TBI and, subsequently, with ABI, with the aim to address EF in everyday settings [43]."<br>"The qualitative data on the process of adaptation of the original TOPS to the Italian context (I-TOPS) is extensively reported as case study in a paper published in 2021 [32]."		
**5-iv) Quality assurance methods**		
"No change on the I-TOPS or wellness intervention content will be made during the trial, therefore both interventions will be delivered in their initial Italian version."		
**5-v) Ensure replicability by publishing the source code, and/or providing screenshots/screen-capture video, and/or providing flowcharts of the algorithms used**		
"To ensure fidelity and quality assurance, all psychologists will receive training in recruitment strategies, interviewing techniques, questionnaire administration and delivery of the I-TOPS and wellness interventions prior to beginning the program implementation"<br>"In relation to the I-TOPS intervention, psychologists will receive weekly supervision from a qualified psychotherapist (Master in Clinical Psychology, specialization in Cognitive Behavioral Psychotherapy, qualification in Clinical Neuropsychology) to ensure treatment fidelity and quality assurance."		
**5-vi) Digital preservation**		
Table 1 reports intervention session content.		
**5-vii) Access**		
http://www.itops.it/ (I-TOPS intervention)<br>https://tops.itops.it/it/login/ (wellness intervention)		
**5-viii) Mode of delivery, features/functionalities/components of the intervention and comparator, and the theoretical framework**		
"Participants will not have to pay or will not be paid to access the allocated intervention."; "Participants' personal data will be pseudonymized."<br>"Each participant will be identified in all study-related documentation by the trial number and initials."<br>"To prevent problems with mislaid usernames and passwords, participants will access the webpages of the allocated training program through links emailed to them by the research team members. Once the program has been completed by the participant, it will be locked to prevent further data entry or change."		
**5-ix) Describe use parameters**		
"The regular version of I-TOPS consists of 10 core sessions focused on problem-solving, EF, behavioral strategies and social skills, and 10 supplemental sessions.The 10 core modules consist of self-guided didactic content regarding the steps of problem-solving and strategies for managing everyday challenges, brief videos modelling these skills and exercises to support implementation."<br>"The program will not provide any feedback on activities done."<br>"After the family has completed and reviewed the contents of each session, the trained psychologist will conduct a video meeting of approximatively one hour with the adolescent and the parent..."<br>"Patients included in the wellness intervention will complete the treatment according to the same schedule as patients of the experimental group, but will not receive direct intervention on EF nor support with respect to the problem-solving process. This control treatment focuses on health and wellness, but omits contents on problem-solving, EF, behavioral strategies and social skills and goal setting."<br>The wellness intervention consists of 10 core sessions and 3 supplemental sessions..."		
**5-x) Clarify the level of human involvement**		
"No recommendations on timing, frequency or intensity of use will be provided; the only requirement will be the completion of each session within a 2 weeks' window."		
**5-xi) Report any prompts/reminders used**		
"Every two weeks a psychologist located in the rehabilitation center (Scientific Institute, IRCCS E. Medea, Bosisio Parini, Lecco, Italy) will contact patients and families using Google Meet videoconference platform to provide remote monitoring, content discussion and support on the problem-solving process for the I-TOPS intervention and remote monitoring and content discussion only for the wellness intervention."		
**5-xii) Describe any co-interventions (incl. training/support)**		
"Prompts to use the training will be provided by emails automatically sent by the program; however, participants will choose the frequency (weekly, bimonthly or monthly) of reminders, by selecting a specific option included in the program website. In addition, bimonthly meetings with the psychologist will review content and promote adherence."		
**6a) CONSORT: Completely defined pre-specified primary and secondary outcome measures, including how and when they were assessed**		
"Inclusion criteria for the study...are:<br>-a diagnosis of non-progressive ABI (TBI, stroke, brain inflammation/infection, anoxia/hypoxia etc.) in the chronic phase (at least one year after the event);<br>-age between 11 and 19 years at the time of the recruitment;<br>-Full scale intellectual quotient ≥ 70<br>-proper comprehension and speaking abilities in Italian;<br>-having a personal computer and access to the Internet in the everyday setting and adolescent and family familiarity with basic computer and Internet literacy to manage emails, access to the Internet and web-sites and video-calls;<br>-at least one parent or guardian living with the adolescent available to participate in the intervention.<br>Exclusion criteria...are:<br>-presence of pre-injury or comorbid conditions, such as sensory impairments and global developmental delay;<br>-a history of abuse;<br>-a history of psychiatric hospitalization;<br>-receiving concomitant psychological intervention."		
**6a-i) Online questionnaires: describe if they were validated for online use and apply CHERRIES items to describe how the questionnaires were designed/deployed**		
**6a-ii) Describe whether and how "use" (including intensity of use/dosage) was defined/measured/monitored**		
The item is not applicable to the manuscript as no online questionnaire was used.		
**6a-iii) Describe whether, how, and when qualitative feedback from participants was obtained**		
"No data on number of logins or average session length will be recorded, as, for both treatments, these data does not provide significant information on better training use and is not considered to be associated with improving target abilities."		
**6b) CONSORT: Any changes to trial outcomes after the trial commenced, with reasons**		
"The I-TOPS and the wellness intervention will be delivered remotely by using a web-based platform including online learning modules."<br>"Every two weeks a psychologist located in the rehabilitation center (Scientific Institute, IRCCS E. Medea, Bosisio Parini, Lecco, Italy) will contact patients and families using Google Meet videoconference platform..."<br>"Demographic, clinical and outcome data will be collected and stored at Scientific Institute, IRCCS E. Medea, Bosisio Parini, Lecco, Italy."		
**7a) CONSORT: How sample size was determined**		
**7a-i) Describe whether and how expected attrition was taken into account when calculating the sample size**		
**7b) CONSORT: When applicable, explanation of any interim analyses and stopping guidelines**		
"A total of 9 outcome measures will be used to test preliminary evidence of training efficacy. Specifically, 7 self-assessed outcome measures (5 questionnaires for parents and 2 questionnaires for adolescents), 2 performance-based tasks assessing social cognition taken from a standardized neuropsychological battery and a virtual-reality based assessment of every-day setting EF will be used."<br>For further information see manuscript, heading "Efficacy outcomes" in the Methods section.		
**8a) CONSORT: Method used to generate the random allocation sequence**		
No change in trial outcomes after the trial commenced is expected.		

**8b) CONSORT: Type of randomisation; details of any restriction (such as blocking and block size)**		
The item is not applicable to the manuscript, as it reports a trial protocol.		
**9) CONSORT: Mechanism used to implement the random allocation sequence (such as sequentially numbered containers), describing any steps taken to conceal the sequence until interventions were assigned**		
"In detail, the randomization will be conducted by a researcher of the Institute, independently from the research staff responsible for recruiting participants. Randomization of patient assignment to the groups will follow a coin flip procedure using the randomization tool of Microsoft Excel: an automated number will be randomly associated to any recruited patient and determine assignment to G1 (0 to 0.49) or G2 (0.50 to 1)."		
**10) CONSORT: Who generated the random allocation sequence, who enrolled participants, and who assigned participants to interventions**		
"...participants will be randomized into one of two groups: G1, receiving the regular I-TOPS treatment, or G2, receiving the active control training, namely the wellness treatment."<br>"No stratification will be used."		
**11a) CONSORT: Blinding - If done, who was blinded after assignment to interventions (for example, participants, care providers, those assessing outcomes) and how**		
**11a-i) Specify who was blinded, and who wasn't**		
**11a-ii) Discuss e.g., whether participants knew which intervention was the "intervention of interest" and which one was the "comparator"**		
"Indeed, to allow for clinical supervision, treatment fidelity, and patient interactions, the supervising psychotherapist will be required to receive group allocation information. Other research staff, participants, and testers will remain blinded to group assignment. Given these considerations, the present study will constitute a double-blinded RCT."		
**11b) CONSORT: If relevant, description of the similarity of interventions**		
"Randomization of patient assignment to the groups will follow a coin flip procedure using the randomization tool of Microsoft Excel: an automated number will be randomly associated to any recruited patient and determine assignment to G1 (0 to 0.49) or G2 (0.50 to 1)."<br>"The independent researcher will give the staff members responsible for the study (A.B. and C.C.) a sealed envelope containing the participant's group assignment to keep it concealed from research staff."		
**12a) CONSORT: Statistical methods used to compare groups for primary and secondary outcomes**		
The manuscript reports the full trial protocol.		
**12a-i) Imputation techniques to deal with attrition / missing values**		
**12b) CONSORT: Methods for additional analyses, such as subgroup analyses and adjusted analyses**		
"The wellness intervention consists of 10 core sessions and 3 supplemental sessions, in order to provide adolescents and their families with a program having the identical structure to the original I-TOPS."		
RESULTS		
**13a) CONSORT: For each group, the numbers of participants who were randomly assigned, received intended treatment, and were analysed for the primary outcome**		
We included major details on the methods adopted		
**13b) CONSORT: For each group, losses and exclusions after randomisation, together with reasons**		
"No interim analysis will be planned."		
**13b-i) Attrition diagram**		
**14a) CONSORT: Dates defining the periods of recruitment and follow-up**		
"...the expected 42 subjects were randomized into the 2 treatment groups and 34 of them concluded the assigned intervention and underwent post-training and 6 month-follow-up evaluations. The total attrition rate was 19%."		
**14a-i) Indicate if critical "secular events" fell into the study period**		
**14b) CONSORT: Why the trial ended or was stopped (early)**		
""...the expected 42 subjects were randomized into the 2 treatment groups and 34 of them concluded the assigned intervention and underwent post-training and 6 month-follow-up evaluations. The total attrition rate was 19%."		
**15) CONSORT: A table showing baseline demographic and clinical characteristics for each group**		
"Recruitment started on December, 27, 2021 and ended on February, 25 2022. Last patient evaluation was performed on February, 22, 2023."		
**15-i) Report demographics associated with digital divide issues**		
**16a) CONSORT: For each group, number of participants (denominator) included in each analysis and whether the analysis was by original assigned groups**		
**16-i) Report multiple "denominators" and provide definitions**		
**16-ii) Primary analysis should be intent-to-treat**		
The item is not applicable to the manuscript as it reports the trial protocol.		
**17a) CONSORT: For each primary and secondary outcome, results for each group, and the estimated effect size and its precision (such as 95% confidence interval)**		
The item is not applicable for the study as the trial was not stopped early.		
**17a-i) Presentation of process outcomes such as metrics of use and intensity of use**		
**17b) CONSORT: For binary outcomes, presentation of both absolute and relative effect sizes is recommended**		
The item is not applicable to the manuscript as it reports the trial protocol.		
**18) CONSORT: Results of any other analyses performed, including subgroup analyses and adjusted analyses, distinguishing pre-specified from exploratory**		
No results for each group on efficacy are reported in the manuscript as it is the trial protocol.<br>"Significance threshold will be set at <0.05."		
**18-i) Subgroup analysis of comparing only users**		
**19) CONSORT: All important harms or unintended effects in each group**		
The item is not applicable to the manuscript as it reports the study protocol and does not include data on efficacy.		
**19-i) Include privacy breaches, technical problems**		
**19-ii) Include qualitative feedback from participants or observations from staff/researchers**		
"Participants' personal data will be pseudonymized. A unique trial number will be provided to each participant consenting to partake in the study"<br>"To prevent problems with mislaid usernames and passwords, participants will access the webpages of the allocated training program through links emailed to them by the research team members."		
DISCUSSION		
**20) CONSORT: Trial limitations, addressing sources of potential bias, imprecision, multiplicity of analyses**		
**20-i) Typical limitations in ehealth trials**		
**21) CONSORT: Generalisability (external validity, applicability) of the trial findings**		
**21-i) Generalizability to other populations**		
**21-ii) Discuss if there were elements in the RCT that would be different in a routine application setting**		

"In addition, the examination of training and study procedure feasibility will allow investigating the usability of the training and the adequateness and potential replicability of the study design, which could help addressing potential issues to intervention delivery in the clinical context and study replication by future research teams. This could also favor the evaluation of the generalizability of trial findings to the general patient population of adolescents with brain injury, addressing sources of potential bias and imprecision."		
**22) CONSORT: Interpretation consistent with results, balancing benefits and harms, and considering other relevant evidence**		
**22-i) Restate study questions and summarize the answers suggested by the data, starting with primary outcomes and process outcomes (use)**		
**22-ii) Highlight unanswered new questions, suggest future research**		
The discussion section does not include interpretations of results, as the manuscript is the trial protocol.		
Other information		
**23) CONSORT: Registration number and name of trial registry**		
The item is not applicable to the manuscript as it reports the study protocol and does not include data on efficacy.		
**24) CONSORT: Where the full trial protocol can be accessed, if available**		
"The risks associated with participating in this trial are considered minimal. The exclusion of adolescents with photosensitive epilepsy reduces the possible risks associated with the prolonged use of a technological device."<br>For further information see manuscript, heading "Safety reporting"<br>The manuscript is the trial protocol, thus no data on unintended effects in each group are reported.		
**25) CONSORT: Sources of funding and other support (such as supply of drugs), role of funders**		
"This study was registered with Clinicaltrials.gov with identifier NCT05169788 on December, 23, 2021."		
**X26-i) Comment on ethics committee approval**		
**x26-ii) Outline informed consent procedures**		
"This study has been approved by the Ethic Committee of Scientific Institute, IRCCS E. Medea, Bosisio Parini, Lecco, Italy (Prot. N. 08/21 – CE, January 21, 2021)."		
**X26-iii) Safety and security procedures**		
"The informed consent will be obtained face-to-face by staff members responsible for the study (A.B. and C.C.) when the adolescent and parents are/come to the clinic or remotely, by using a sealed envelope, for those families not able to reach the Institute."		
**X27-i) State the relation of the study team towards the system being evaluated**		
